# Experience of disruption of capsule endoscopy after prolonged retention

**DOI:** 10.1002/deo2.57

**Published:** 2021-09-29

**Authors:** Yohei Furumoto, Akihiro Araki, Taichi Matsumoto, Takahito Nozaka, Masato Yauchi, Katsumasa Kobayashi, Sayuri Nitta, Eriko Okada

**Affiliations:** ^1^ Department of gastroenterology Tokyo Metropolitan Bokutoh Hospital Tokyo Japan; ^2^ Department of Health Management Center Toranomon Hospital Tokyo Japan; ^3^ Department of gastroenterology and Hepatology Tokyo Medical and Dental University Tokyo Japan

**Keywords:** bowel diseases, capsule endoscopy, case report, double‐balloon enteroscopy, intraoperative complication

## Abstract

Capsule endoscopy is an effective tool for evaluating small bowel diseases. Capsule retention is a complication of capsule endoscopy, but capsule disruption after retention has not been thoroughly studied. Only a few cases of capsule disruption have been reported. We report a case of capsule disruption after prolonged retention. A 73‐year‐old woman underwent capsule endoscopy for the evaluation of anemia. One week later, capsule retention was observed on radiography. Capsule removal was advised, but she refused because she did not have any symptoms. After 20 months, computed tomography revealed disrupted capsule fragments. Capsule removal was strongly recommended, and the patient agreed. All disrupted capsule fragments were removed using double‐balloon endoscopy without complications. Intestinal perforation had been prevented by removing the disrupted capsule before the battery fluid leaked into the intestinal tract. Capsule retention, documented by imaging, should be addressed by removing the retained capsule immediately before capsule disruption occurs.

## INTRODUCTION

Capsule endoscopy (CE) allows for direct noninvasive examination of the small bowel and has become the gold standard for evaluating small bowel diseases. Capsule retention is a complication of CE.^1^ However, capsule disruption after retention rarely occurs and has not been studied thoroughly. Only a few cases of disruption of CE have been reported. CE disruption increases the risk of intestinal perforation. Thus, retained CE should be performed immediately. However, the interval between capsule retention and disruption is unknown. We report a case of CE disruption after prolonged retention and recommend measures for capsule disruption. This study was conducted according to the principles of the Declaration of Helsinki. Verbal informed consent was obtained from the patient for publication of detail of the case.

## CASE REPORT

**FIGURE 1:**
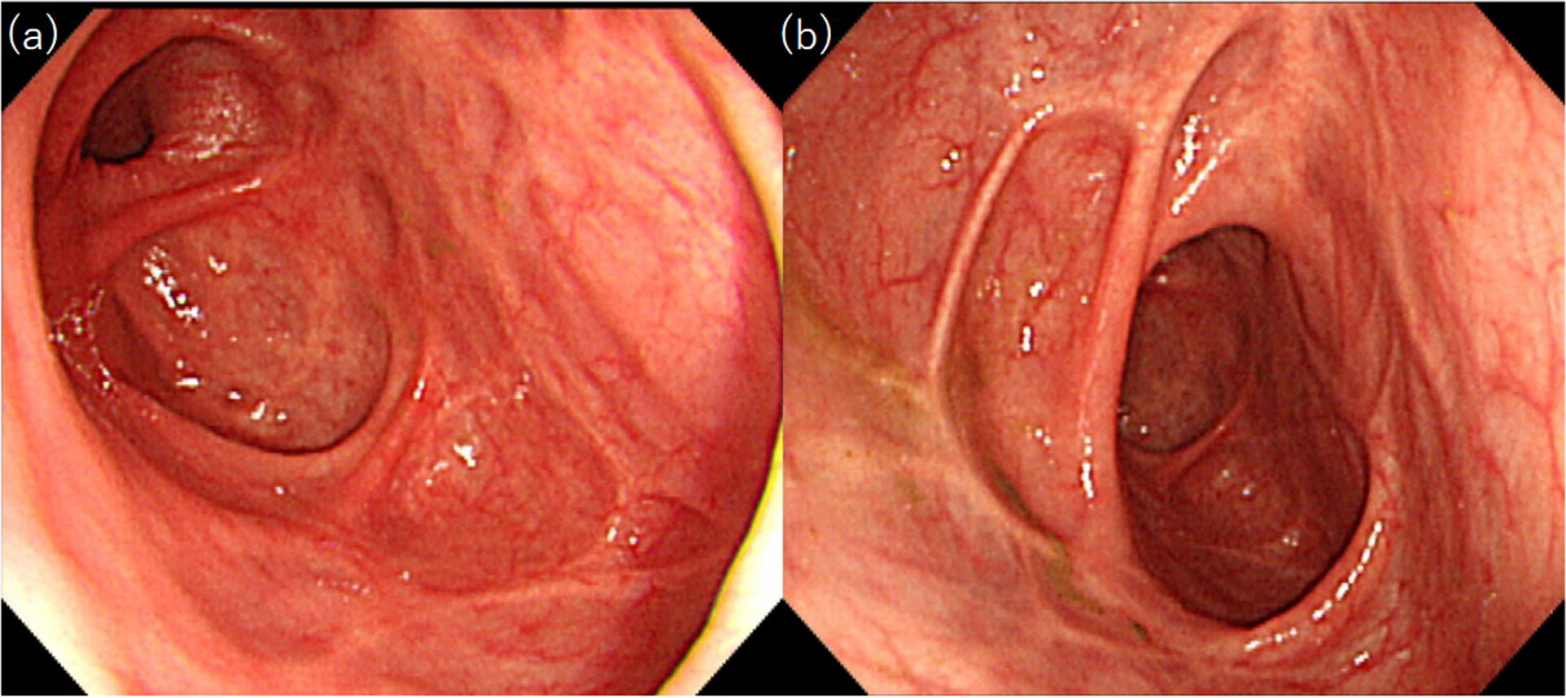
Multiple, deformed, ulcer scars were observed in the cecum and ascending colon (a and b)

A 73‐year‐old woman was referred to our gastroenterology department by her family doctor for evaluation of anemia. She had a history of hypertension, cerebral infarction, intestinal tuberculosis, and abdominal surgery for a perforated duodenal ulcer 46 years ago. She did not have a history of Crohn's disease or neoplastic lesions. Her current medications included a nonsteroidal anti‐inflammatory drug and low‐dose aspirin. Physical examination revealed conjunctival pallor, spoon nails, and edema of the lower extremities. Laboratory tests revealed iron deficiency anemia. She had a hemoglobin of 3.9 g/dl, ferritin of 8 μg/dl, an unsaturated iron‐binding capacity of 394 μg/dl, a total iron‐binding capacity of 402 μg/dl, and ferritin of 7.8 ng/ml. Esophagogastroduodenoscopy and colonoscopy were performed to evaluate the bleeding site, but the cause of the anemia was not identified. However, multiple deformed, ulcerated scars suspicious of intestinal tuberculosis were observed in the cecum and ascending colon (Figure 1a,b). We explained to the patient the need for CE for additional evaluation of the cause of anemia as well as the risk of capsule retention and the possible need for endoscopic or surgical removal. Written informed consent to CE was obtained from the patient. We had attempted to assess gastrointestinal (GI) patency using a patency capsule (PC) before CE. However, because the patency capsule was not discharged within 33 h, the patency was evaluated using abdominal radiography. Since we determined that the PC had reached the sigmoid colon, CE was performed. CE showed multiple mucosal erosions. No other abnormal findings were observed.

**FIGURE 2:**
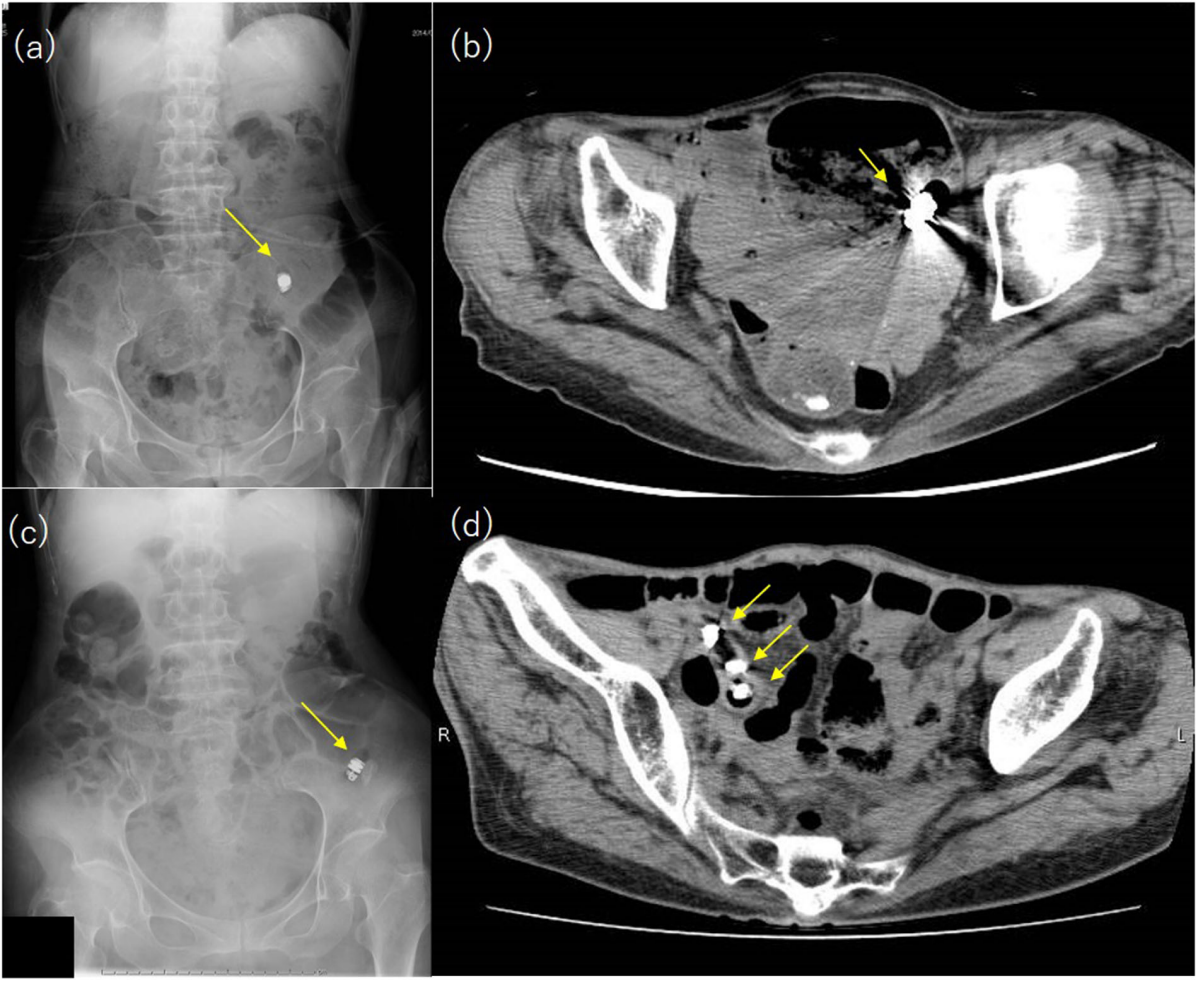
Radiography (a) and computed tomography (CT) (b) images taken one week after capsule endoscopy revealed retention of a capsule. Radiography images after 18 months revealed the existence of a capsule, which was not disrupted yet (c). CT image taken 20 months after capsule endoscopy revealed a fragmented capsule. The capsule was divided into three components (d) (arrows)

**FIGURE 3:**
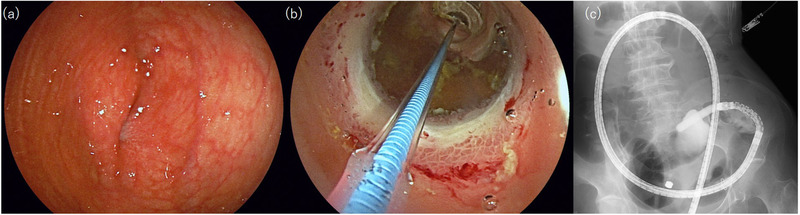
Endoscopic image: the stenotic lesion of the ileum before dilation using a balloon catheter (a). Endoscopic (b) and radiographic images (c) the stenotic lesion was dilated using a balloon catheter

**FIGURE 4:**
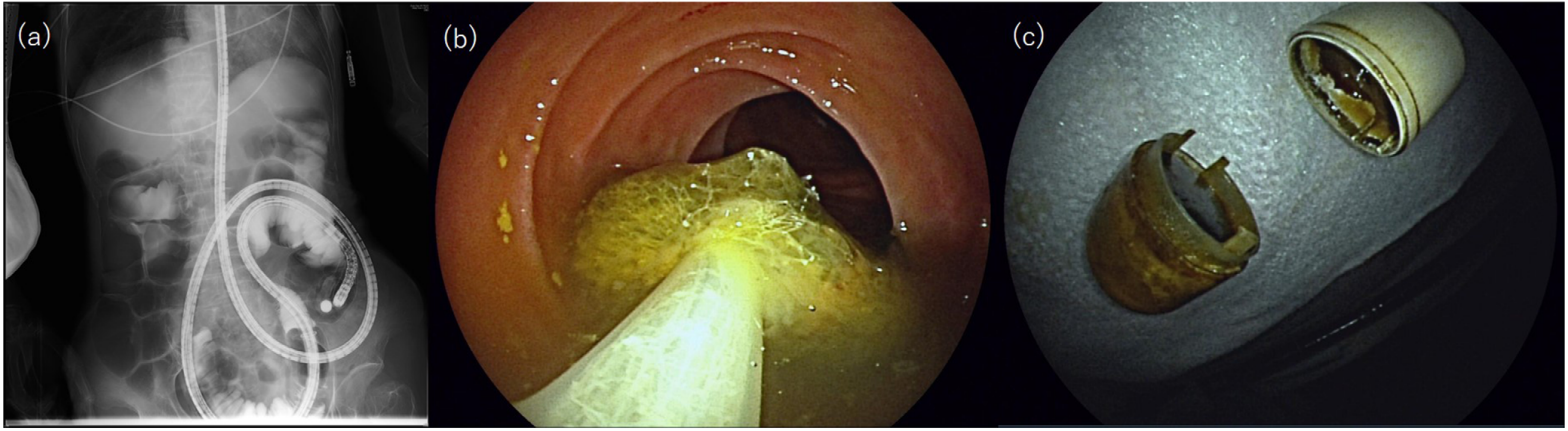
Double balloon endoscopy (DBE) reached the position of the capsule fragments (a). DBE captured all the pieces of the fragmented capsule using a retrieval net (b). Removed capsule fragments: The batteries were contained within the capsule (c)

One week later, the CE had not yet been discharged. Abdominal radiography and computed tomography (CT) scans revealed the presence of a capsule (Figure 2a,b). Capsule removal was advised, but the patient refused because she did not experience any symptoms. Abdominal radiography was subsequently performed monthly for four months. During this period, an intact capsule was observed on radiography. After the 4 months, abdominal radiography or CT was performed every 3–6 months. Abdominal radiography revealed an intact capsule at 12 and 18 months (Figure 2c). At 20 months, CT revealed a fragmented capsule that was divided into three pieces (Figure 2d). Capsule removal was strongly advised, and the patient agreed. At 27 months, double‐balloon endoscopy (DBE) was performed. Initially, retrograde DBE showed multiple annular ulcer scars with stenosis in the ileum. Because the DBE could not pass the three stenosed scar lesions, they were dilated using a balloon catheter (Figure 3a,c). We inserted the DBE as deep as possible, however, it could not reach the location of the disrupted capsule fragments. Subsequent antegrade DBE showed several annular ulcer scars with stenosis. Because the DBE could not pass the stenotic lesion, it was dilated. The DBE reached the position of the disrupted capsule fragments (Figure 4a), and the fragments, which included two batteries, were captured using a retrieval net (Figure 4b,c). The batteries were contained within the capsule and did not induce intestinal tract complications, such as perforation.

Although serological, histopathological, and culture findings specific to intestinal tuberculosis were not found due to the patient being in the healing period, we suspected a previous episode of intestinal tuberculosis because of the multiple annular ulcer scars in the ileum. The multiple deformed ulcer scars in the cecum and ascending colon, and the history of intestinal tuberculosis.

## DISCUSSION

We report a case of CE disruption after retention for 20 months. All disrupted capsule fragments were successfully removed using DBE.

Capsule disruption after retention is a rare complication of CE. Only three cases of capsule disruption have been reported (Table 1).^2^, ^3^, ^4^ A disrupted capsule should be removed immediately because the battery fluid, whose electrochemical effect can cause intestinal perforation, can leak out of the capsule. In previous cases, open abdominal surgery was performed to remove the capsule fragments. Surgical intervention has been the most common approach for the retained capsule removal. However, recent reports have recommended the use of endoscopic treatment.^5^, ^6^ DBE was selected to remove the CE in this case. The removal of disrupted capsule fragments by DBE has not been previously reported.

**TABLE 1:** Reported cases of capsule endoscopy disruption after retention

	**Year**	**Authors**	**Age**	**Sex**	**Previous diagnosis**	**Periods of retention**	**Symptom**	**Treatment**
1	2005	Fry et al.^2^	76	M	No abdominal disease	6 months	Abdominal pain	Open abdominal surgery
2	2011	de Magalhaes Costa et al.^3^	53	F	Crohn's disease	3.3 years	None	Open abdominal surgery
3	2014	Royall et al.^4^	39	M	Crohn's disease	3 years	Abdominal pain	Open abdominal surgery
4	2021	Our case	73	F	Intestinal tuberculosis	2.3 years	None	Double balloon endoscopy

The rate of capsule retention in CE has varied from 0% to 13%, depending on the indications for examination.^1^ The longest duration of capsule retention was 4.5 years. In this case, the patient did not exhibit symptoms during capsule retention.^7^ Similarly, in our case, the patient did not exhibit any symptoms, so she opted out of capsule removal. Thus, the capsule was retained for 2.3 years. The periods of retention in previous cases of capsule disruption ranged from 6 months to 3 years. Since the shortest interval between capsule retention and disruption was 6 months only, the retained CE should be removed immediately after documentation with abdominal radiography or CT scans.

In addition, preventing capsule retention is essential. Initially, it was necessary to listen to detailed medical history. History of Crohn's disease, abdominal surgery, small bowel obstruction, and chronic nonsteroidal anti‐inflammatory drug use predisposed the patient to capsule retention.^1^ In this case, the patient had a history of intestinal tuberculosis and abdominal surgery. Therefore, the risk of capsule retention was high. Prior assessment of GI patency significantly minimized the risk of capsule retention. The patency capsules were reportedly safe and efficacious for evaluating intestinal strictures. It can be used before conventional CE to predict and minimize the risk of retention.^8^, ^9^ When a PC is not discharged within the defined time, an imaging modality must be selected to identify its position. Abdominal radiography is commonly chosen, but it is often difficult to identify whether a PC is in the small intestine or colon. When evaluation of GI patency is challenging, continuing with additional investigations can help improve the procedure's safety. In our case, we attempted to assess GI patency using a PC before CE; however, since only abdominal radiography was performed as a further investigation, we incorrectly determined that the PC had reached the sigmoid colon while it was retained in the small intestine. Additional evaluations would have led to an improved assessment of GI patency. While CT can detect a PC with a higher probability than radiography, the increased radiation exposure is a concern. The effectiveness of low‐dose CT, which helps reduce radiation exposure, has been reported.^10^

Capsule disruption is a rare complication. We reported a case of capsule disruption after CE. Since capsule disruption can cause intestinal perforation by battery fluid leakage, disrupted capsule fragments should be removed immediately.

We successfully retrieved the disrupted capsule fragments by DBE without complications. DBE was a viable strategy for removing disrupted capsule fragments.

## CONFLICT OF INTEREST

The authors declare that they have no conflict of interest.

## FUNDING INFORMATION

None.
